# Looking (for) patterns: Similarities and differences between infant and adult free scene-viewing patterns

**DOI:** 10.16910/jemr.13.1.2

**Published:** 2020-04-01

**Authors:** Daan R. van Renswoude, Maartje E. J. Raijmakers, Ingmar Visser

**Affiliations:** University of Amsterdam, The Netherlands; Free University, Amsterdam , The Netherlands

**Keywords:** Infant eye movements, Scene perception, Ambient and focal processing, Systematic tendencies, scan path, eye tracking, saccades

## Abstract

Systematic tendencies such as the center and horizontal bias are known to have a large influence on how and where we move our eyes during static onscreen free scene viewing. However, it is unknown whether these tendencies are learned viewing strategies or are more default tendencies in the way we move our eyes. To gain insight into the origin of these tendencies we explore the systematic tendencies of infants (3 - 20-month-olds, N = 157) and adults (N = 88) in three different scene viewing data sets. We replicated com-mon findings, such as longer fixation durations and shorter saccade amplitudes in infants compared to adults. The leftward bias was never studied in infants, and our results indi-cate that it is not present, while we did replicate the leftward bias in adults. The general pattern of the results highlights the similarity between infant and adult eye movements. Similar to adults, infants’ fixation durations increase with viewing time and the depend-encies between successive fixations and saccades show very similar patterns. A straight-forward conclusion to draw from this set of studies is that infant and adult eye movements are mainly driven by similar underlying basic processes.

## Introduction

Within real-world scenes there is always more visual information available than we can process. This implies that we must selectively allocate our attention towards different parts of the scenes. When looking at a real-world scene our eye movements are series of fixations and saccades. During a fixation we process the available visual information and the saccades are the re-locations towards other parts of the scene. The planning of these eye movements is influenced by both bottom-up scene characteristics (1) and top-down cognitive relevance (2). There are systematic tendencies in the way we move our eyes, such as the central (3,4) and horizontal bias (5,6). These systematic tendencies could be a result of bottom-up or top-down influences, or could be a result of other unrelated processes. In this paper we take a developmental perspective by comparing systematic tendencies in eye movements of infants and adults.

Systematic tendencies are often interpreted as confounding factors obscuring the effects of bottom-up or top-down processes. As such the term ‘biases’ is often used to refer to these tendencies. Tatler and Vincent (7) showed that predicting fixation locations based on systematic tendencies alone (e.g., blind to the scene) can outperform a saliency map based on low-level image features (e.g., contrast, luminance, edges). In order to conclude that bottom-up or top-down processes play a role in guiding eye movements, this influence should be above and beyond of what is expected based on systematic tendencies alone. In the analyses of eye movement data much effort has gone into deriving appropriate baseline measures to control for the influence of these systematic tendencies (4,8) and accounting for these tendencies in analysis models (9,10). Recently, Clarke, Stainer, Tatler, and Hunt (11) developed the saccadic flow model which provides a baseline measure to control the influence of fixation locations on saccade directions.

Apart from being a confounding factor, these systematic tendencies are also used to improve the prediction of fixation locations in saccadic models. Saccadic models aim to predict fixation locations by generating series of fixations and saccades known as scanpaths. These saccadic models can be improved by incorporating these systematic tendencies. For instance, Le Meur and Coutrot (12) improved their earlier saccadic model (13) by incorporating the joint distribution of saccade lengths and directions. More recently they also showed that adding these age-related tendencies into the model improved the prediction of fixation locations within the different age groups (14).

Although these systematic tendencies are a robust phenomenon observed across experiments, tasks and labs, the nature of these tendencies remains largely unknown. Comparing infants and adults with regard to these tendencies as we do in the current study, allows to gain more insight into the origin of these tendencies. Infants’ ability to make saccadic eye movements is present from birth. Newborns are already able to follow a moving object using saccadic eye movements (15) and the ability to track objects using smooth pursuit movements develops from 2 to 5 months of age (16,17). In order to perceive static real-world scenes infants also require perceptual functions such as, contrast sensitivity, depth perception and color discrimination. These functions are limited at birth, however they develop rapidly during the first few months of life. Although development continues until early childhood, around 3-4 months of age these functions are developed sufficiently well to perceive colorful real-world scene with high acuity (18). All in all, the necessary capacities of the visual system of infants are developed sufficiently well to make meaningful comparisons with adult eye movements during static scene perception.

There are a couple of explanations where these systematic tendencies originate from. First, we could be “hard wired” to move our eyes in certain ways that causes these dependencies. This could be both a result of physical constrains, such as the distribution of rods and cones in the retina (19) or be a result of a core knowledge module (20) that determines how to move our eyes. Second, these systematic tendencies could also be the result of the scene content. There are known spatial dependencies in scenes (21) that may cause these systematic behaviors. Third, these tendencies could be the result of top-down strategies that we use to explore the environment. There is some evidence that relationships between successive fixations and saccades are different for free-viewing tasks, compared to search tasks. For instance, Nuthmann (9) reports a stronger relationship between incoming saccade amplitude and fixation durations for a search task than a free-viewing task. This may indicate that these systematic tendencies are affected by cognitive strategies. Fourth, these tendencies may be learned as being an effective or efficient way to process a scene. If these systematic tendencies are similar in infants and adults the physical and scene content explanations become more likely. Similar to adults, infants use both low-level visual saliency and high-level features such as faces and objects to guide their attention during scene viewing. In addition, although the eyes continue to mature during development, physical differences in the eye movement system are relatively small between infants and adults. If, however, there are differences between infants and adults in their systematic tendencies, it would be more likely that cognitive factors or learning play a role as these are factors that infants have not fully developed yet.

### Systematic tendencies

The overall systematic tendencies, such as the center bias and the horizontal bias are also known to be present in infants (22, 23, 24), however the existence of other systematic tendencies is largely unknown. The current study examines the leftward bias, viewing time and scan path dependencies between successive fixations and saccades which are described below in more detail.

### Leftward bias

The leftward bias is the tendency to make an initial eye movement towards the left side of the screen (25, 26, 27). This tendency is hypothesized to stem from the asymmetry of attentional control networks in the brain that are lateralized to the right hemisphere. Another common explanation is the reading direction that is left-to-right for most participants and that these learned scanning habits play a role in the leftward bias. Although the leftward bias seems universal, studies comparing left-to-right readers with not left-to-right readers report a weaker leftward bias for non-left-to-right readers (28, 29, 31). If reading direction does influence this tendency, we would not observe the leftward bias in infants, but we would in adults.

### Viewing time

The influence of viewing time on eye movements is well-established. At the start of a trial, fixation durations are typically shorter after which they increase in duration during the trial (9,32,33). Saccade amplitudes tend to follow an opposite pattern with larger saccade amplitudes at the start of the trial that decrease in amplitude towards the end (34,35). Together these findings have been interpreted to reflect different modes of scanning. An ambient or global mode at the start of trials characterized by shorter fixation durations and saccades of longer amplitude and a focal or local mode with longer fixation durations and saccades of shorter amplitude (34). However, Follet, Le Meur, and Baccino (36) reported an initial occurrence of the focal mode followed by an ambient mode, based on their findings they suggest there is an interplay between the modes during viewing. Having different modes of viewing behavior could be interpreted as strategic viewing, a quick scan to identify the most informative regions followed by a closer inspection of this region.

### Scan path dependencies

Given that fixation duration and saccade amplitude depend on viewing time, we would also expect dependencies between successive fixation durations and saccade amplitudes if the ambient and focal modes exist (34). Similarly we would also expect correlations between successive fixation durations and successive saccade amplitude. Tatler and Vincent (35) did report such dependencies in which shorter fixation durations and saccade amplitudes were also followed by shorter fixation durations and saccade amplitudes and longer fixation durations and saccade amplitudes were followed by longer fixation durations and saccade amplitudes. Other scan path dependencies include the relationships between fixation durations and saccade amplitudes with the (change in) saccade direction.

### Current study

The main aim of the current study is to investigate the origin of the systematic tendencies often reported in the literature. To this end we compare infants and adults on the systematic tendencies frequently observed in adults in three existing free-viewing data sets. Studying infants in comparison with adults allows to gain more insight in the mechanisms underlying the systematic tendencies. When results are similar for infants and adults it is more likely these tendencies are a result of basic mechanisms, whereas differences between adults and infants could indicate that these tendencies are a result of more elaborate cognitive strategies used by adults or are learned over time.

A secondary aim is to describe these systematic tendencies in infants, such that researchers studying infant viewing behavior can control the influence of these tendencies and avoid getting biased results. To explore the similarities between systematic tendencies in infants and adults during free scene viewing we will examine the leftward bias, the effects of viewing time and the scan path dependencies between successive fixations and saccades. For this last part we will closely follow (35) who examined these systematic tendencies in adults.

## Methods

This is an exploratory study in which we re-analyze three data sets from previous scene viewing studies with infants and adults. The original studies examined the horizontal bias (23), the center bias (22) and object familiarity (37). Throughout this paper we will refer to these study by these names. The original papers include a detailed description of the participants, materials and procedure, here we provide a brief description.

### Participants

Table 1 shows the main descriptives of the participants in the three studies. Combined 157 infants (M = 9.71 month-olds, range = 3.20 - 20.53) and 88 adults (undergraduate psychology students) saw around 30 photographs of real-world scenes while their eye movements were recorded. All studies were conducted in accordance with the declaration of Helsinki and all adult participants and infant caretakers gave their informed consent.

**Table 1:** Number of participants (N), number of trials (n), time per trial in seconds, total number of fixations (Nfix) and the mean age of the infants and adults in the three studies.

Study	Group	N	Males	n (time per trial)	Nfix	Mean age (sd, min-max)
Center	Adults	20	-*	n = 30, (2.5 s)	2368	20.79 Years (2.17, 18-24)
bias	Infants	50	22	n = 30, (2.5 s)	2662	12.25 Months (4.02, 5.46-20.53)
Horizontal	Adults	48	20	n = 28, (4.0 s)	11092	21.40 Years (4.89, 17-39)
bias	Infants	52	19	n = 28, (4.0 s)	5181	8.99 Months (3.58, 3.20-15.47)
Object	Adults	20	7	n = 29, (8.0 s)	9672	22.30 Years (1.58, 18-25)
familiarity	Infants	55	30	n = 29, (8.0 s)	11198	9.43 Months (2.21, 5.93-13.06)

*gender was not registered for adults in this study

### Materials

**Figure 1.**
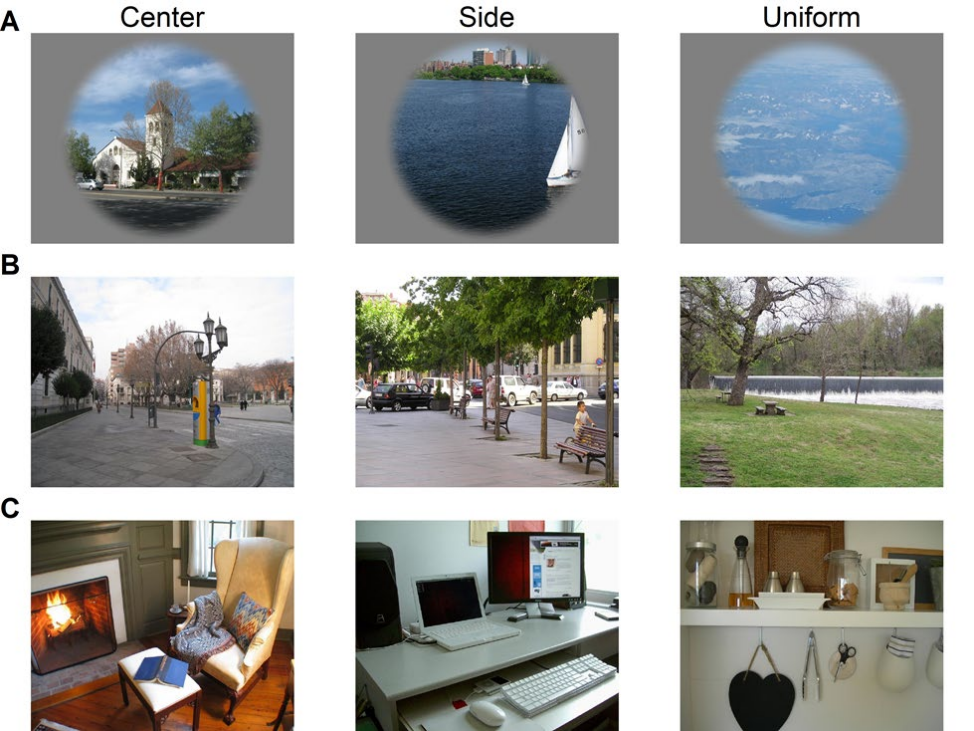
Examples of the stimuli used in the *center bias* study (row A), *horizontal bias* study (row B) and *object familiarity* study (row C).

For the center bias study a total of 30 stimuli were selected with specific requirements for three conditions (i.e. 10 stimuli in each condition). Stimuli either had saliency distributions biased towards the center, biased towards the side, or uniformly distributed saliency distributions, see Figure 1 row A. In the original study we manipulated the start position and our main interest was the first saccade. For this re-analysis the first fixation is excluded to limit the effect of the manipulation. Another large difference between the center bias study and the other two studies was the layout of the stimuli. Stimuli were presented overlaid with a circular aperture to avoid directional biases due to screen dimensions. In the horizontal bias study 28 stimuli were selected from the labelme database (38) and in the object familiarity study 29 real-world images were selected from the Object and Semantic Images and Eye-tracking (OSIE) data set (39), for examples see Figure 1 row B & C. In these studies the presentation time was 4 and 8 seconds respectively and stimuli were presented full screen (object familiarity) or with a black border around the stimuli while maintaining the aspect ratio of the screen.

### Procedure

In all studies, eye movements were recorded using a remote-optics corneal reflection eye-tracker (SR EyeLink 1000), with a sampling rate of 500Hz. Visual stimuli were presented on a 17-inch monitor (1280x1024) in full color extending approximately 34° x 27° of visual angle, such that the number of pixels per degree of visual angle was approximately 38. After participants were properly seated approximately 60 centimeters from the computer monitor in either a Maxi-Cosi or on their caregiver lap, lights were dimmed and black curtains were drawn such that only the stimuli presented on the computer monitor could be seen. Caregivers were instructed not to communicate with the infant or to (re)act on the images presented on the screen. A 5-point calibration scheme was used in all studies and the experiment began once the mean error of all calibration points was smaller than 1 degree of visual angle. The calibration used colorful looming dots or cartoons accompanied with sounds to attract the infants attention. The center bias and object familiarity studies started based on a gaze-contingent attention getter between each trial. If this attention getter was not fixated 5 times in a row, there was an option to re-calibrate if the attention getter was missed due to drift from the original calibration. In the horizontal bias study the trials started with a fixation cross and no re-calibration took place during the experiment.

### Manuscript preparation

This manuscript is prepared in Rstudio using R (40) and the R-packages cowplot (41), gazepath (42), ggforce (43), ggplot2 (44), gridExtra (45), and papaja (46) for all analyses and visualizations.

### Data analysis

The current study is an explorative study and as such we do not report inferential statistics or use statistical tests. The way we do present the data is using visualizations that include confidence intervals. Based on these confidence intervals conclusions can be drawn based on the similarities or differences between infants and adults. Overlapping confidence intervals implies there most likely is no significant difference, whereas non overlapping intervals indicate possible differences between infants and adults.

## Results

### Data descriptives

**Figure 2.**
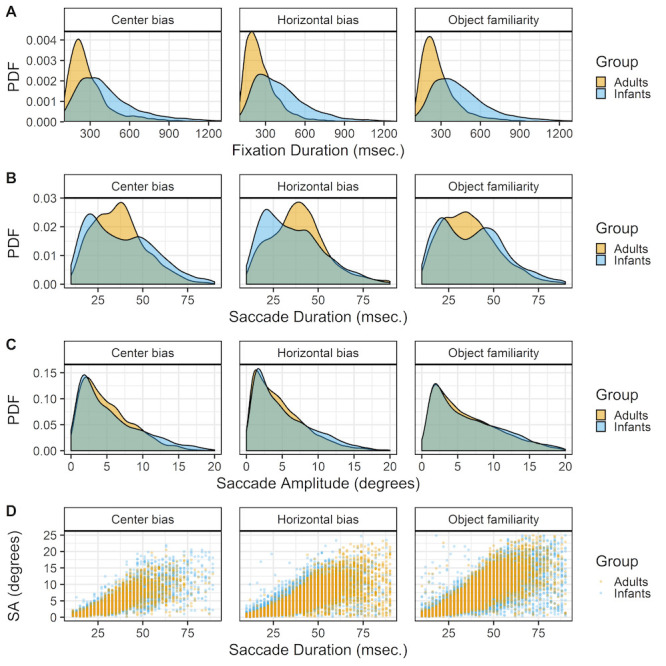
The probability density functions (PDFs) smoothed with a Gaussian kernel of fixation durations (row A), saccade durations (row B) and saccade amplitude (row C). The scatter plots on the bottom (row D) show the relationships between the saccade durations and saccade amplitudes (SA). All panels show the data of infants and adults, the three columns show the data of the center bias, horizontal bias and object familiarity study respectively.

Fixations. For all studies the raw data is classified into fixations with the gazepath R-package (42). This method allows to identify fixations in both infant and adult data by setting individual thresholds such that noisier data results in more conservative thresholds. Since infant eye-tracking data is typically noisier than adult eye-tracking data this method is suitable to compare both groups as the same method can be used while allowing differences within individual. The top row of Figure 2 shows the densities of fixation durations for the infants and adults in the three studies. There is a clear pattern that fixation durations are longer in infants than adults.

 Saccades. The gazepath method is optimized to identify fixations and not saccades. However, since the goal of this study is to get a better insight in the characteristics underlying gaze patterns, the saccades are also of interest as gaze patterns are a series of fixations and saccades. To identify the saccades the time between consecutive fixations was calculated. In a typical gaze pattern this is the duration of the saccade, however the period in between fixations can also reflect blinks or missing data instead of a saccade. As saccades typically last between 10 and 90 msec. (47), these values were used as cut-off values to identify saccades.

There are clear differences between the durations of saccades of infant and adults. Saccades of both adults and infants seems to follow a bi-modal distribution with modes around 20 and 40 msec., however the mode around 40 msec. is more prominent in adults whereas the mode around 20 msec. is more common in infants (Figure 2 row B). These differences in the durations are only to a small extent reflected in the amplitudes, Figure 2 row C. This difference may stem from the gazepath method with which the saccades were identified. The gazepath method sets individual speed thresholds based on the amount of noise in the data. In the noisier infant data the thresholds are set higher than in the less noisy adult data. This implies that the start of adult saccades is identified earlier than the start of infant saccades, whereas the end of adult saccades is identified later than the end of infant saccades. This differences in threshold may very well explain the differences in saccade duration between infants and adults and also explains why this difference is almost non-existent for the saccade amplitudes. The saccade amplitudes are calculated as the distance between fixations and those distances are not affected by the different thresholds for infants and adults. It is thus important to be careful in interpreting these differences in saccade durations as they may very well be a result of the different thresholds. On the other hand, these small differences cannot be completely ignored as they also seem to be present in the saccade amplitude data, albeit to a much smaller degree. These differences may reflect some sort of developmental pattern in which adults are more likely to make larger saccades than infants. This is in line with findings of others comparing infants (24) and children (48) with adults during a scene viewing task.

Although the use of individual speed thresholds in the gazepath method may exaggerate differences between infants and adults in saccade durations, the bi-modality of the saccade durations cannot be explained as a by-product of the saccade identification methods. The bi-modality of saccade durations in both infants and adults also exists when the standard Eyelink classification method is used, is reported by others for scene viewing tasks (47), and is also observed in other experimental tasks in our lab. This bi-modality in saccade durations may reflect different processing modes (34) in which scene exploration is focal (resulting in short and small saccades) or ambient (resulting in long and large saccades). This would imply that the bi-modality is also present in the saccade amplitude data, but this is not clearly visible by only looking at the distribution.

### Leftward bias

**Figure 3.**
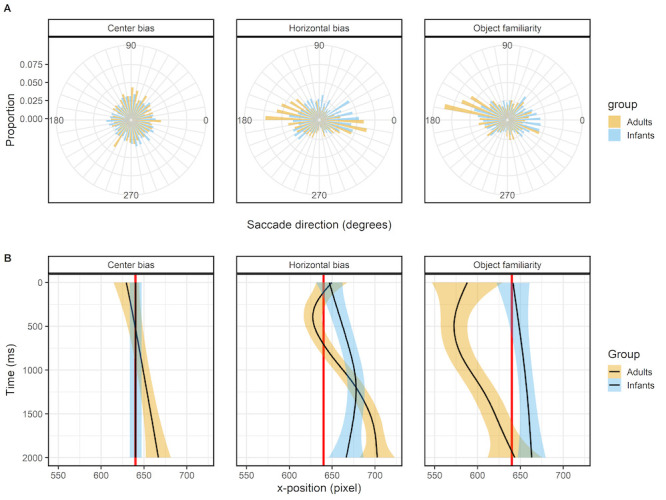
The top panels (row A) show the saccade directions of the first saccade. The bottom panels (row B) shows the mean x-coordinate, the shaded areas correspond to the 95% confidence intervals calculated using the Generalized Additive Models (49) method with default values as implemented in the geom_smooth function of the R-package ggplot2 (44). All panels show the of infants (blue) and adults (yellow).

Figure 3 shows the histogram of the proportions first saccade directions in the top panels (row A) and the mean x-position of the fixations as a function of time in the lower panels (row B). For the center bias data set there is no leftward bias, this can both be seen in the histogram of proportion in which the saccade directions are evenly distributed as in the x-position as a function of time where the mean x-position of both infants and adults’ fixations does not deviate from the center of the screen (red line). This is a very sensible outcome as the start position was manipulated in this study, which also explains the wide range of x-positions at the start of the trial. Therefore it shouldn’t be expected that there would be a leftward in this data set.

For the horizontal bias data set there is a clear bias in the horizontal directions as can be seen from the histogram of the proportions, it can also be seen that the leftward bias is stronger in adults than infants. The initial shift of the x-position for adults is to the left, but based on the confidence interval does not seem to deviate much from the center of the screen (red line). Both infants and adults seem to have an overall rightward bias for the horizontal bias data set, which may explain why we didn’t replicate earlier findings. The object familiarity data set does show a very clear leftward bias for adults, but not for infants in both the histogram of saccade directions and the x-position. There is bias in adults to target the first saccade towards the (top) left, which results in shift in the x-position of fixations that is also biased to the left. In infants the direction of initial saccades is much more evenly distributed and as such there is also no overall leftward bias in the x-position. Overall, the pattern of results in these three studies can be taken as evidence that adults have a leftward, but only when the start position is at the center, whereas infants do not have a leftward bias.

### Viewing time

**Figure 4.**
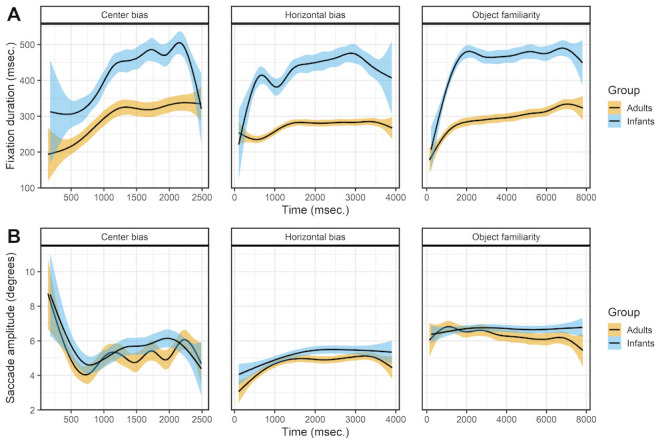
The top panels (row A) show the saccade directions of the first saccade. The bottom panels (row B) shows the mean x-coordinate, the shaded areas correspond to the 95% confidence intervals calculated using the Generalized Additive Models (49) method with default values as implemented in the geom_smooth function of the R-package ggplot2 (44). All panels show the of infants (blue) and adults (yellow).

Figure 4 shows the effect of viewing time on the fixation durations (row A) and saccade amplitudes (row B). The fixation durations of both infants and adults show a sharp initial increase after which the fixation durations stabilizes or keep increasing slightly. For adults these patterns are in line with earlier studies examining the effect of viewing time on fixation durations (9,33,34). Infants show a similar pattern as adults, which is a similar result as reported by Helo et al. (24) who also compared infants and adults. They found that fixation durations made early during the trial were shorter than fixations durations made later during the trial for older infants (> 9 months) and adults, but there was no difference for younger infants.

The effect of viewing time on saccade amplitudes is similar for infants and adults but without a clear pattern across data sets, see Figure 4B. In the center bias data set there is a sharp initial decrease which is the result of the manipulated start position after which most saccades were made towards the center. Afterwards there seems to be a small increase in saccade amplitudes with viewing time. This is similar to the pattern found in the horizontal bias data set, which also shows a slight increase, but in the object familiarity data set there is no effect of viewing time on saccade amplitudes. These mixed results are not in line with a decrease in saccade amplitude during viewing (33,34), however they do matched the adult data reported by Follet et al. (36) and are also similar to the effects Helo et al. (24) report for both infants and adult.

### Scan path dependencies

**Figure 5.**
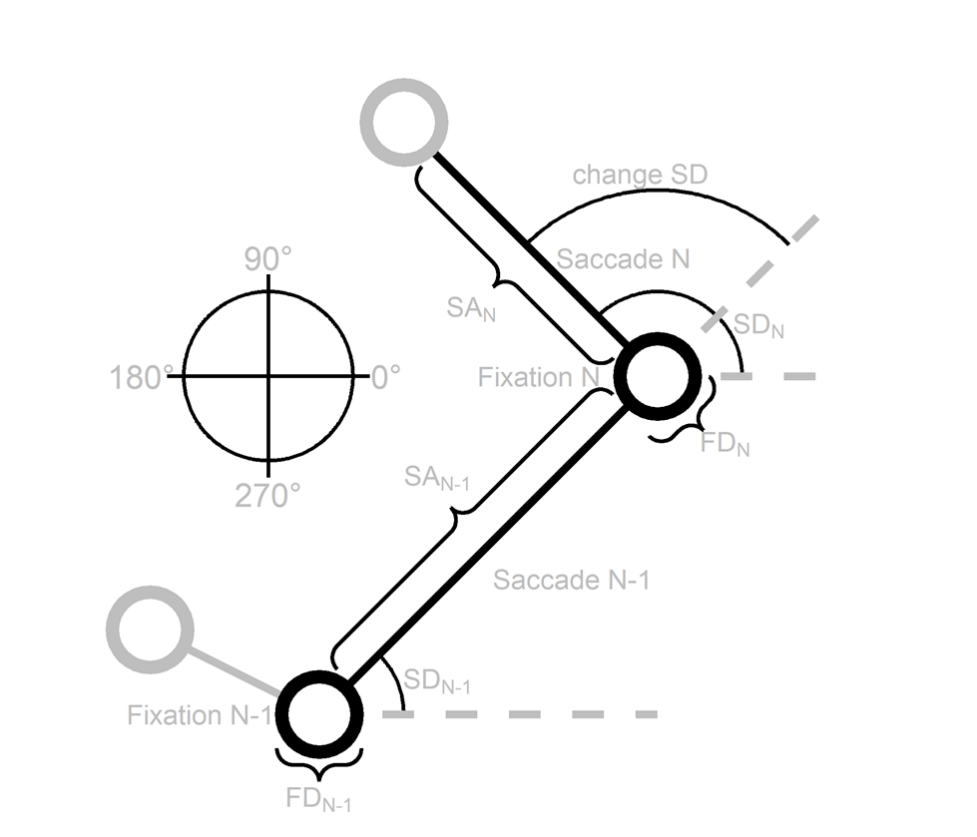
A schematic scan pattern that shows how the previous (N-1) and current (N) fixation duration (FD), saccade amplitude (SA), saccade direction (SD) and change in saccade direction (change SD) are defined. The direction rose on the left shows how the saccade directions can range from 0 to 360 degrees where saccades to the right are 0 degrees, upward saccades 90 degrees, saccades to the left 180 degrees and downward saccades 270 degrees. The layout of this Figure is adapted from Tatler and Vincent (2008).

To get a better understanding of the relationship between successive fixations and saccades we adopt the same method as (35) and examine dependencies between the previous (N-1) and current (N) fixation duration (FD), saccade amplitude (SA), saccade direction (SD) and change in saccade direction (change SD), see Figure 5. This Figure displays a schematic scan patterns to show how the different variables are defined. In the following sections the dependencies between these variables are examined.

### Previous and current saccade amplitudes

**Figure 6.**
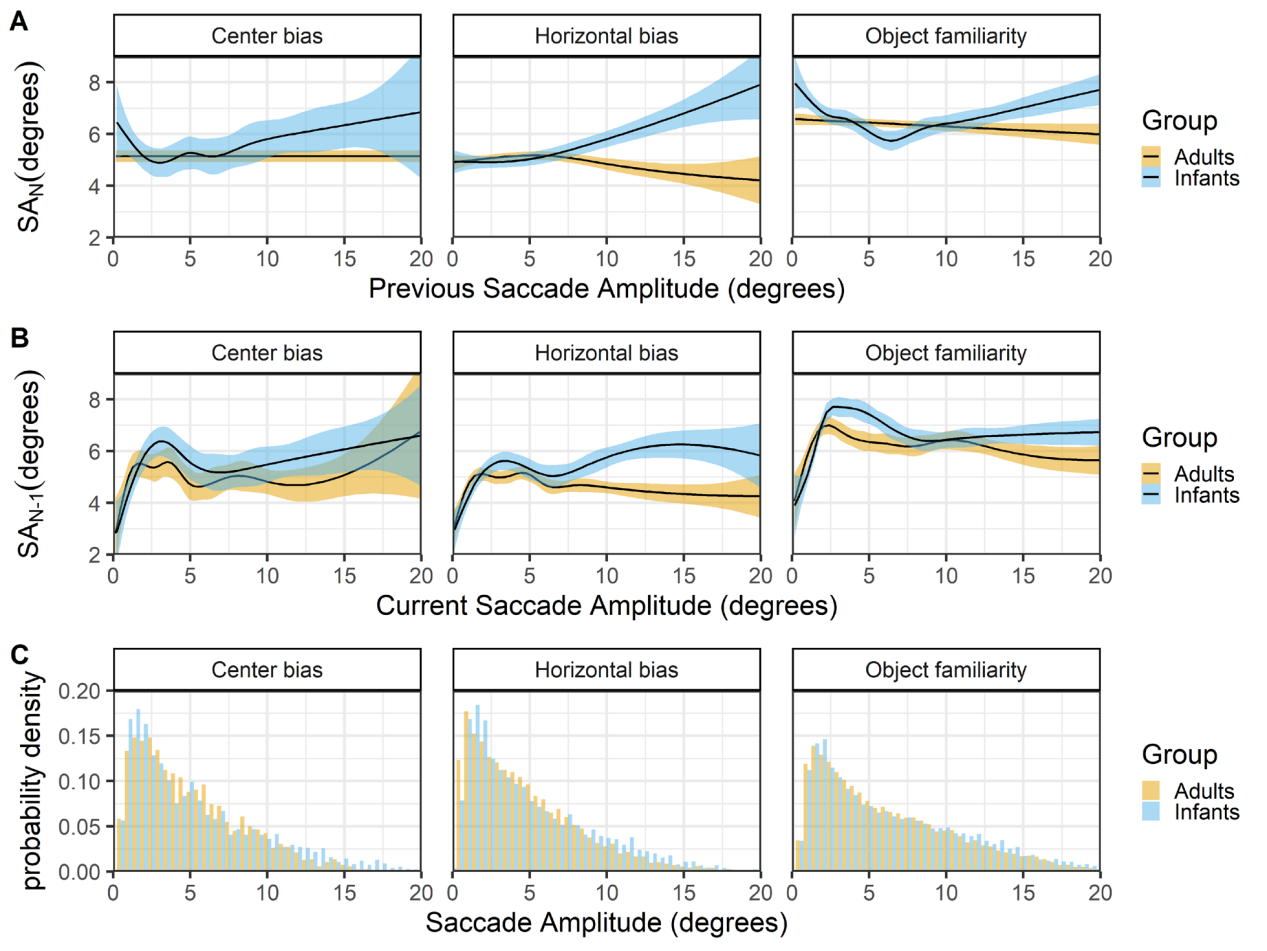
The top panels (row A) show the previous saccade amplitude (SA_(N-1)) as a function of the current saccade amplitude (SA_N). The middle panels (row B) show the current saccade amplitude (SA_N) as a function of the previous saccade amplitude (SA_(N-1)). These functions are fitted with 99% confidence intervals using the Generalized Additive Models (49) method with default values as implemented in the geom_smooth function of the R-package ggplot2 (44). The bottom row (C) shows the probability density of the saccade amplitudes for infants (blue) and adults (yellow).

Figure 6 shows the relationship between successive saccades (rows A & B) and the histogram of saccade amplitudes for infants and adults. The top panels (row A) show that infants and adult differ in their saccadic behavior. In adults large amplitude saccades are more likely to be followed by a smaller amplitude saccade, whereas infants large amplitude saccades are more likely to also be followed by a larger amplitude saccade. The finding in adults are relatively consistent across data sets (expect for the center bias data) and replicate the relationship reported by (35). When the current saccade amplitude is used as predictor (row B), the relationship between infant and adult successive saccades looks similar both within and across the different data sets. There is positive relationship in which small saccades are also preceded by small saccades, this could indicate periods of local scanning (34) in both infants and adults. For larger saccades there is a trend in which larger amplitude saccades are preceded by smaller saccades in adults, replicating earlier work (35). In infants there is also an initial decline (between 3-8 degree saccades) in which larger amplitude saccades are preceded by smaller saccades, but for larger saccades this seems to shift towards a positive relationship.

### Previous and current fixation durations

**Figure 7.**
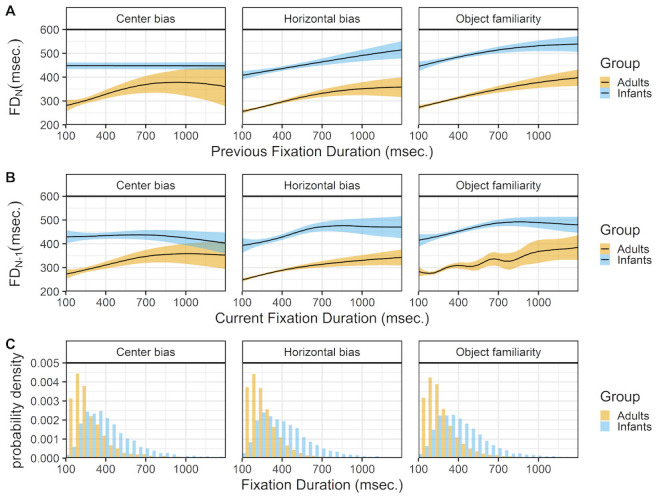
The top panels (row A) show the previous fixation duration (FD_(N-1)) as a function of the current fixation duration (FD_N). The middle panels (row B) shows the current fixation duration (FD_N) as a function of the previous fixation duration (SA_(N-1)). These functions are fitted with 99% confidence intervals using the Generalized Additive Models (49) method with default values as implemented in the geom_smooth function of the R-package ggplot2 (44). The bottom row (C) shows the probability density of the fixation durations for infants (blue) and adults (yellow).

Figure 7 shows the relationship between successive fixations (rows A & B) and the histogram of fixation durations (row C) for infants and adults. In adults the overall pattern is very similar between studies. Longer fixations are more likely to be followed or preceded by longer fixations. In infants the overall pattern is the same for the horizontal bias and object familiarity data sets, but there is no influence of preceding or successive fixation duration in the center bias data. Again these results are strikingly similar to the results reported by Tatler and Vincent (35).

### Fixation duration and saccade amplitudes

**Figure 8.**
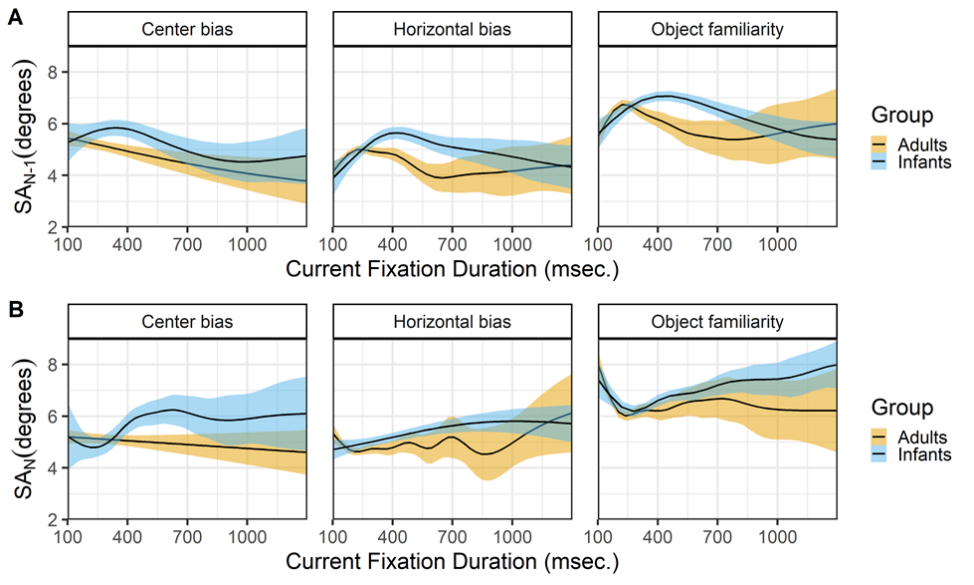
The top panels (row A) show the previous saccade amplitude (SA_(N-1)) as a function of the current fixation duration (FD_N). The bottom panels (row B) show the current saccade amplitude (SA_N) as a function of the current fixation duration (FD_N). These functions are fitted with 99% confidence intervals using the Generalized Additive Models (49) method with default values as implemented in the geom_smooth function of the R-package ggplot2 (44).

Figure 8 shows the relationship between the current fixation duration and incoming (row A) and outgoing (row B) saccades. The top panels (row A) show a very similar pattern for infants and adults across the different data sets. Except for adults in the center bias data, both infants and adults show an inverted U-shape relationship between the current fixation duration and the incoming saccade amplitude. Shorter and longer fixation durations tend to be preceded by saccades of smaller amplitude, while fixation durations around 200 msec. in adults and 400 msec. in infants tend to be preceded by saccades of larger amplitude. These peaks of the inverted U-shape corresponds to the median fixation durations in both infants and adults. Again the relationship for adults closely matches the relationship reported by Tatler and Vincent (35).

The relationship between the current fixation duration and the amplitude of the outgoing saccade is somewhat different for both infants and adults and across data sets (row B). The 2 most similar patterns are the patterns of adult in the horizontal bias and object familiarity data sets. Saccade amplitudes are large after short fixation duration and this relationship disappears for longer fixation durations. These results are very much in line with the results reported by others (34,35). Do note that they also report a strong positive relationship for fixations durations shorter than 100 milliseconds, that we cannot assess as we only consider fixation durations of 100 milliseconds and longer. The infants in the object familiarity data set show a similar pattern as adults, although there seems to be an increase in which longer fixations tend to be followed by saccades of larger amplitude. This patterns of longer fixations followed by larger saccades is also present for infants in the other two data sets.

### Saccade directions

**Figure 9.**
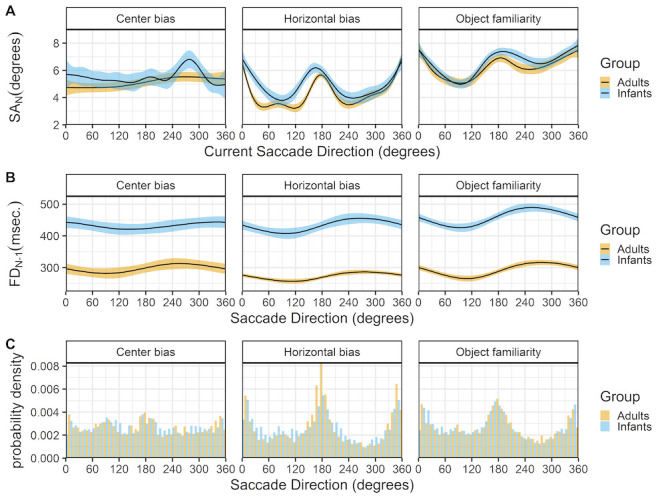
The top panels (row A) show the current saccade direction (SD_N) as a function of the current saccade amplitude (SA_N). The middle panels (row B) show the current saccade direction (SD_N) as a function of the previous fixation duration (FD_(N-1)). These functions are fitted with 99% confidence intervals using the Generalized Additive Models (49) method with default values as implemented in the geom_smooth function of the R-package ggplot2 (44). The bottom panels (row C) shows the probability density of the saccade directions for infants (blue) and adults (yellow).

Figure 9 shows the relationship between the current saccade direction and current saccade amplitude (row A) and preceding fixation duration (row B). The horizontal bias and object familiarity data sets show the typical horizontal bias for infants and adults, however this bias is not present in the center bias data set, see Figure 9C. A possible explanation is the layout of stimuli in the center bias study were circular, which is known to influence the bias (50). In addition, the manipulation of the start position and the selection of the stimuli in different conditions most likely has influenced the overall horizontal bias.

There is a strong relationship between the saccade direction and saccade amplitude in both infants and adults (row A). Saccades along the horizontal axis are longer, followed by downward saccades which have an intermediate amplitude, while the upward saccades have the smallest amplitude. Again these findings are strikingly similar for both infants and adults and are very much in line with reports by others (35). The middle panels (row B) show a similar relationship between the saccade direction and the preceding fixation duration for infants and adults. Downward saccades are preceded by shorter fixations, while upward saccades are preceded by longer fixations, the saccades to the left and right fall in middle and are preceded by fixations with an intermediate duration. Interestingly these relationship seems also to be present in the adult data of the center bias data set, despite the lack of an overall horizontal bias effect. Again this effect is also reported by Tatler and Vincent (35).

### Change in saccade directions

**Figure 10.**
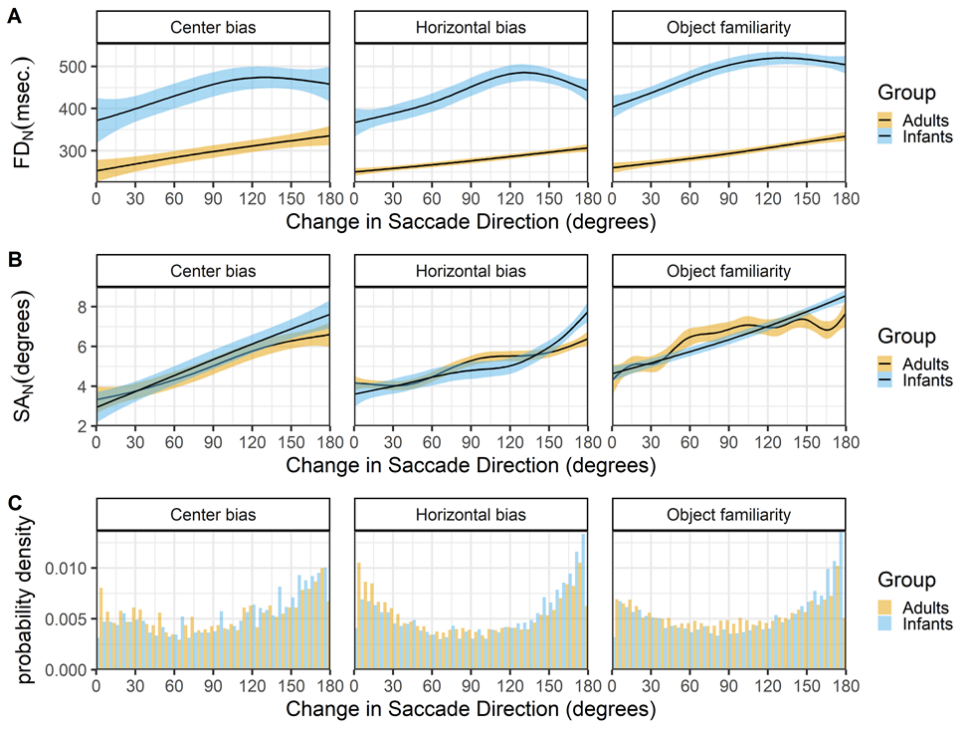
The top panels (row A) show the change in saccade direction as a function of the current fixation duration (FD_N). The middle panels (row B) show the change in saccade direction as a function of the current saccade amplitude (SA_N). These functions are fitted with 99% confidence intervals using the Generalized Additive Models (49) method with default values as implemented in the geom_smooth function of the R-package ggplot2 (44). The bottom panels (row C) show the probability density of the change in saccade directions for infants (blue) and adults (yellow).

Figure 10 shows the relationship between the change in saccade direction and current fixation duration (row A) and current saccade amplitude (row B). All data sets show a remarkable similar pattern for both infants and adults. Larger changes in saccade direction co-occur with longer fixation durations (row A) and larger saccade amplitudes (row B). These findings have also been reported by others (9,35) and thus seem very robust.

## Discussion

The main aim of the current study was to investigate the origin of the systematic tendencies in eye movements over real-world scenes. Therefore we explored the leftward bias, the effects of viewing time and the scan path dependencies in both infants and adults. Overall, the results show that infants have very similar systematic tendencies in their eye movements as adults. Moreover, the systematic tendencies we found in adults, almost entirely replicated results reported by others with regarding to the leftward bias (26,27), scan path dependencies (9,35) and viewing time (24,33,34). The results were also very similar across the three data sets, with the exception of the center bias data set in which the results of the saccade directions, saccade amplitudes and the leftward bias did differ from the other two data sets. These exceptions can be explained by the experimental setup in which the start position was set away from the center, the scenes were divided in three categories and the viewing time was only 2.5 seconds. The fact that many systematic tendencies are still present in this data sets, is another example of the robustness of these tendencies. 

The largest difference between infants and adults was found with regard to the leftward bias. This bias is not present in infants, but we did observe the leftward bias in adults replicating earlier studies (25, 26, 27). Brain asymmetries related to attentional control are an explanation for the bias (26), but the results of this study indicate that reading scanning habits may also play a role. Ossandon et al. (26) argued that the reading explanation is less likely as they observed that handedness influenced the leftward bias, which fits better with the brain asymmetry explanation than the reading explanation. Moreover, they note that the leftward bias is also observed in infants and animals looking at faces (51). However, the effects of handedness did not replicate in a recent study (27) and the current study does not find any leftward bias effects in infants. The leftward bias for faces is frequently reported (52, 53, 54) and may be specific to faces due to the right hemisphere dominance in face processing. In addition, the initial saccade bias observed in the current study is not straight to the left, but to the left and up, as would be expected if the leftward bias is a scanning habit learned from reading. Taken together, it seems plausible that reading may play a role in the leftward bias.

The effects of viewing time on fixations durations and saccade amplitudes did not completely match with the idea of ambient and focal processing modes. Unema et al. (34) theorized that scene viewing is characterized by an initial ambient mode when the scene is scanned with short fixations and long saccades, followed by a focal mode with longer fixations and shorter saccades. Here we did observe the increase in fixation durations with viewing time (9,55), but not the decrease of saccade amplitudes over time reported by others (33,34). However, the effects of saccade durations on viewing time having been also reported to increase with viewing time (24,55,56). The fact that we did observe mixed effects seems to fit in with the mixed results reported in the literature. Given the pattern of results described in other studies and the current study it seems unlikely that scene viewing is characterized by an initial ambient mode, followed by a focal mode. However, ambient and focal modes may still exists, albeit independent of viewing time.

The effects that successive saccades have within scan paths corroborate the existence of a focal mode, but not of an ambient mode. Replicating (35), we observed that saccades of short amplitude are often preceded by saccades of short amplitude, but for longer saccades there was no or a minimal effect. This observation is in line with a focal mode in which one region is inspected closely leading to successive short saccades, but if there would be an ambient mode we would also have observed a (strong) relationship for longer saccades. For the fixation duration we did observe that shorter fixation durations are followed by shorter fixation durations and longer fixation durations are followed by longer fixation durations. However, this effect may also be an artifact of the fixation durations that increase with time. All in all the effects of successive fixations and saccades are robust across studies and age groups and seem to suggest that a focal scanning mode exists.

Another remarkable robust finding is the effect of outgoing saccade direction on fixation durations. Saccades in the upward direction are preceded by shorter fixation durations than saccades in the horizontal directions and downward saccades are preceded by the longest fixation durations (35). Fixation duration are often assumed to reflect processing speed (57,58), which is also a common explanation for the (much) longer fixation duration in infants than in adults (10,24). However, these results suggest that at least a part of the fixation durations in both infants and adults is influenced by similar processes related to saccade planning. This effect can have important implications in more experimental designs. Researchers using saccadic task, for instance the gap-overlap task (59), anticipation tasks (60) or spatial negative priming task (61), in which saccadic reaction times are the dependent variable should be well aware of this effect.

The effect of saccadic momentum can also be a confounding factor in experimental studies that rely on saccadic reaction times. Here we found that fixations between saccades that continue in the same direction are shorter than fixations between saccades in which the direction changes. This is also a robust finding for both infants and adults, found across data sets and also reported in adult studies (9,35). In addition the change of saccade direction also influenced the saccade amplitude, saccades in the same direction are shorter than saccades in the opposite direction. This effect may very well be an artifact of the fixed scene size: after moving your eyes in one direction there is simply less scene left to further move your eyes in the same direction and more space to move in the opposite direction, it is therefore not surprising that this effect is very similar for infants and adults.

Overall the systematic tendencies described in this study are very similar for infants and adults. This is quite a remarkable finding as adult eye movements are often assumed to be driven by cognitive relevance (2,62) and/or more elaborate scanning strategies such as having a ambient and focal mode (34). Moreover, most of the effects reported in this study directly replicate what others report (9,27,34,35). Given the replication crises in psychology (63) it is remarkable that the systematic tendencies reported in this study are robust effects that exist independent of country, lab, age group, eye tracking device, etc.

It is common to use these systematic tendencies to improve models that predict fixation locations (7,14,64). The results presented in this study can help to further improve these types of models. In addition to improving prediction, the findings of the current study can help to explain how and when we move our eyes. The systematic tendencies reported in this study can be thought of as default tendencies during free-viewing and set a benchmark for future studies. Studying how we deviate from these default tendencies as a result of experimental manipulations may help to understand the processes that underlie our eye movements. As these tendencies reflect underlying attentional processes, further trying to understand and explain these tendencies can help to move attentional theories forward. As such these default tendencies can be predicted by models of attentional control in order to explain the underlying processes. The current study sets a first step in showing that these underlying processes are likely to be very basic, as the observed tendencies are highly similar for infants and adults.

### Ethics and Conflict of Interest

The author(s) declare(s) that the contents of the article are in agreement with the ethics described in http://biblio.unibe.ch/portale/elibrary/BOP/jemr/ethics.htmland that there is no conflict of interest regarding the publication of this paper. 

### Acknowledgements

This research was supported by the research priority area YIELD, University of Amsterdam, The Netherlands.
